# Supplementary figures and images for: PK-DB: pharmacokinetics database for individualized and stratified computational modeling
Source: Nucleic Acids Res. 2020 Nov 5;49(D1):D1358–64. doi: 10.1093/nar/gkaa990 (PMC7779054; doi:10.1093/nar/gkaa990)

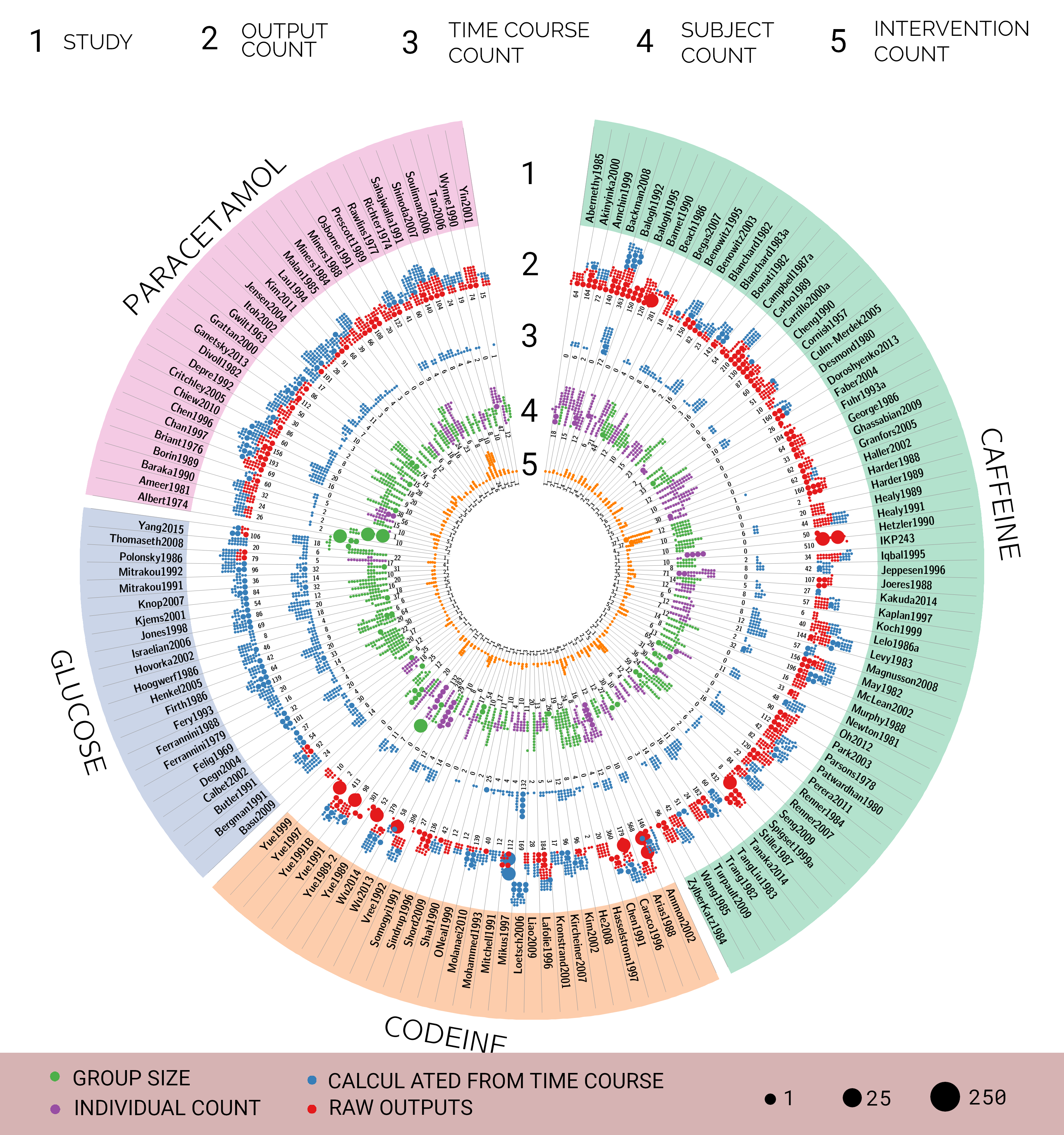

Supplement: gkaa990_Supplemental_Files [file gkaa990_supplemental_files.zip › NAR-sup1.png]

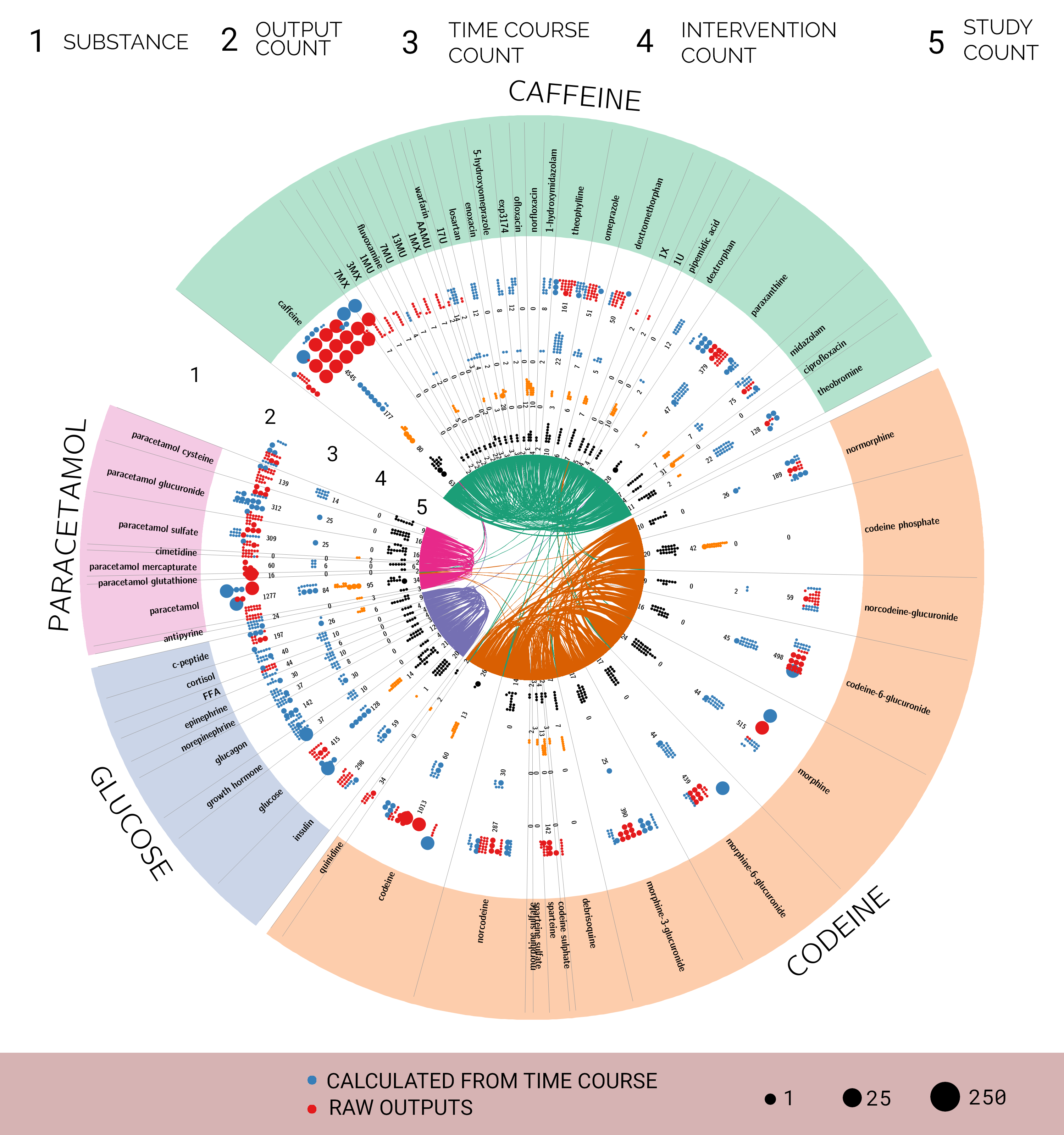

Supplement: gkaa990_Supplemental_Files [file gkaa990_supplemental_files.zip › NAR-sup2.png]
